# Identification of Potential Hazards Associated with South Korean Prawns and Monitoring Results Targeting Fishing Bait

**DOI:** 10.3390/pathogens12101228

**Published:** 2023-10-10

**Authors:** Gyoungsik Kang, Won-Sik Woo, Kyung-Ho Kim, Ha-Jeong Son, Min-Young Sohn, Hee Jeong Kong, Young-Ok Kim, Dong-Gyun Kim, Eun Mi Kim, Eun Soo Noh, Chan-Il Park

**Affiliations:** 1 Department of Marine Biology and Aquaculture, College of Marine Science, Gyeongsang National University, Tongyeong 53064, Republic of Korea; gyoungsikkang@gmail.com (G.K.);; 2 Biotechnology Research Division, National Institute of Fisheries Science, Busan 46083, Republic of Korea

**Keywords:** fishing bait, WSSV, AHPND, diseases of prawns, multiple infection

## Abstract

This study detected two potential pathogens, *Vibro parahaemolyticus*, which causes acute hepatopancreatic necrosis disease (AHPND), and white spot syndrome virus (WSSV), in fishing bait in South Korea. However, their infectious nature was not confirmed, possibly due to the degradation caused by freezing/thawing or prolonged storage under frozen conditions. While infectivity was not confirmed in this study, there is still a significant risk of exposure to these aquatic products. Furthermore, fishing bait and feed should be handled with caution as they are directly exposed to water, increasing the risk of disease transmission. In Australia, cases of WSSV infection caused by imported shrimp intended for human consumption have occurred, highlighting the need for preventive measures. While freezing/thawing is a method for inactivating pathogens, there are still regulatory and realistic issues to be addressed.

## 1. Introduction

The global seafood trade is on the rise, and with the increasing distribution of seafood, the importance of quarantine inspection has also emerged to prevent the spread of diseases. In particular, since the emergence of COVID-19, quarantine inspections are considered almost the only measure for managing infectious diseases. Furthermore, there has been an increasing trend in which each country’s government agencies related to seafood inspection also notify the WTO/SPS of emergency inspection measures for emerging infectious diseases [1,2].

The risk and actual cases of disease transmission caused by seafood products such as fishing bait (regardless of whether quarantine measures are implemented or not) have been mentioned since ancient times [3]. This issue has remained a persistent concern in the field of academia. Therefore, for seafood products that are not subject to quarantine, the risk of disease transmission can be considerably higher. Recently, there have been numerous occurrences of disease transmission caused by fishing bait or feed [4,5,6,7]. The aquatic organism product used as fishing bait or feed is not subject to quarantine inspection in South Korea, which increases the risk of introducing various pathogens. In addition, since infected individuals are immediately exposed to aquatic environments, the risk of disease transmission is very high. Due to these risks, countries have developed various management strategies for seafood used as bait or feed [2,8,9]. In particular, although a variety of species are cultured for use as fishing bait, shrimp and crab are major bait items in South Korea [10].

Prawns, which are primarily used as a food source for human consumption in addition to fishing bait, haave steadily increased in consumption since the early 2000s [11]. However, it is estimated that several tons of prawns die each year due to various diseases [12]. Pathogens that cause significant damage to shrimp include white spot syndrome virus (WSSV), infectious hypodermal and haematopoietic necrosis virus (IHHNV), and Taura syndrome virus (TSV), among others, and relatively recently reported diseases such as covert mortality nodavirus (CMNV), Decapod iridescent virus 1 (DIV1), and Laem-Singh virus (LSNV) also exist in addition to traditional pathogens [12,13,14,15]. In particular, there have been recent cases of detection of WSSV and IHHNV in fishing bait [16] and examples of significant damage caused by pathogens in natural aquatic environments. Therefore, in this study, surveillance was conducted on diseases that could be transmitted to shrimp through aquatic organism products used as fishing bait to investigate diseases that could be hazardous factors for prawns.

The importance of seafood as a major source of protein and economic value cannot be overstated. However, the increasing demand for seafood has led to various challenges, including the spread of infectious diseases among aquatic organisms, which can have severe consequences on the environment, the economy, and human health. In this regard, the transmission of diseases caused by fishing bait and feed is a growing concern that needs to be addressed. Despite the risks associated with the use of aquatic organism products as fishing bait, there is a lack of surveillance and management strategies for diseases that can be transmitted to seafood through fishing bait. Therefore, this study aims to investigate and identify potential hazardous diseases that could be transmitted to prawns through aquatic organism products used as fishing bait. The results of this study can help inform the development of management plans and surveillance programs to prevent the spread of diseases caused by fishing bait and feed, thus ensuring sustainable seafood production and consumption.

## 2. Materials and Methods

### 2.1. Samples Preparation

#### 2.1.1. Sampling

In this study, fishing bait (crustaceans and mollusks), whose origins were verified by the seller, was purchased and utilized for analysis. Every effort was made to secure samples from diverse regions within South Korea (Table 1). To determine the species of the samples, 16S rRNA sequencing was performed according to the methodology of [17], which involves molecular biological identification and identification of the correct scientific name.

#### 2.1.2. Preparation of Tissues for Analysis

When feasible, dissected samples including hepatopancreas, gill, muscle, pleopods, and subcuticular epithelial tissue were extracted for analysis. In cases of severe degradation, the entire cephalothorax was homogenized and utilized for analysis. In instances where the size of the prawn was small, the entire sample was used for analysis.

### 2.2. Identification of Hazardous Factors of Imported Prawns

The present study involved the identification and classification of potential pathogens that may enter South Korea through the import of designated aquatic organisms for quarantine purposes. The identification of hazards was based on ❶ the scientific literature and statistical data, ❷ case studies from other countries that have experienced risks associated with import routes and quarantine-designated items, ❸ expert opinions from fisheries-related and import risk analysis fields, and ❹ aquatic organism disease pathogens designated by the World Organization for Animal Health (WOAH).

Risk factors were determined using the following criteria: ❶ the relevance to quarantine-designated items, ❷ the distribution of susceptible species in South Korea and their potential for causing severe diseases, ❸ the presence of the pathogen in exporting countries, and ❹ the management of the disease by the competent authorities of the exporting country (reporting of disease outbreaks, operation of control and eradication systems). Furthermore, if the pathogen was present in South Korea, hazard factors were selected based on ❶ whether the pathogen in the exporting country was highly pathogenic, and ❷ whether there are areas in South Korea where the pathogen does not occur or occurs at low levels.

Finally, hazard identification was conducted to determine the potential for diseases of prawns to be introduced as a pathogen that negatively affects domestic aquatic life or aquatic ecosystems and the environment due to the import of specific designated quarantine items.

### 2.3. Molecular Biological Analysis

#### 2.3.1. DNA Extraction

Genomic DNA from the tissues from target organs was extracted using AccuPrep^®^ Genomic DNA Extraction Kit (Bioneer, Daejeon, Republic of Korea) following the manufacturer’s guidelines. However, in the case of *Artemia franciscana*, samples were pooled in 0.1 g units due to their small size and used for analysis.

#### 2.3.2. Conventional PCR for Pathogen Detection and Real-Time PCR for Viral Copies

WOAH’s Aquatic Manual or internationally recognized test methods were used for primers used in the experiment (Table S1). A verified synthetic plasmid was used as a positive control, and the target band was confirmed through electrophoresis on 1.5% agarose gel.

For quantitative analysis, a positive control plasmid was constructed, diluted 10-fold, and then a standard curve was prepared using qPCR. The quantitative analysis was followed by protocols of [18].

#### 2.3.3. Definitive Diagnosis

When the target band was confirmed as a result of PCR analysis, a definitive diagnosis was made through Sanger sequence analysis. Before sequence analysis, amplicon was extracted using a QIAquick^®^ gel extraction kit (Qiagen, Hilden, Germany), following the manufacturer’s protocol. The purified PCR products were cloned into pGEM^®^ T-easy vector (Promega, Madison, WI, USA) and transformed into Escherichia coli JM109 competent cells according to the standard protocol. After full propagation, plasmid DNA was extracted using a Hybrid-Q™ plasmid rapidprep kit (GeneAll^®^, Seoul, Republic of Korea) and sequenced using the universal M13 primer set. Nucleotide sequence matching was performed for the identification of pathogen using the basic local alignment search tool (BLAST) algorithm of the National Centre for Biotechnology Information (https://blast.ncbi.nlm.nih.gov/Blast.cgi (accessed on 31 December 2022)).

### 2.4. Histopathological Analysis

Histopathological analysis was performed on the pathogen detected samples. Each sample was fixed in 10% neutral-buffered formalin for 24 h. After fixation, all samples were collected and refixed in the same solution (10% neutral-buffered formalin) for 24 h before being gradually dehydrated with the ethanol series (70–100%). The samples were further cleared with xylene, then embedded in paraffin and sectioned into slices of 4 μm thickness. Finally, the sections were stained with hamatoxylin-eosin (H&E) following general protocols. Stained samples were examined under an optical microscope (Leica DM2500, Wetzlar, Germany).

### 2.5. Transmission Electron Microscopy (TEM) Analysis

For the ultramicroscopic observation of the pathogens in samples, 1 mm^3^ of the frozen samples were fixed in 2.5% glutaraldehyde, refixed in 2% osmium tetroxide, embedded in epoxy (EMS Embed 812, Electron Microscopy Sciences, Hatfield, Montgomery County, PA, USA), dehydrated through a graded acetone series, sectioned to 80 nm, and stained with uranyl acetate and lead citrate. The sections were observed using a transmission electron microscope (Thermo Scientific Talos L120C, Waltham, Middlesex County, MA, USA).

### 2.6. Infectiousness Assessment

To confirm the activity and pathogenicity of the pathogen, an experimental infection was performed using the detected sample. Among pathogens identified as hazardous factors, pathogens that can be cultured were preferentially cultured. In the case of viral pathogens without a separate cell line, mortality was observed for two weeks by directly feeding the detected tissue.

## 3. Results

### 3.1. Sample Acquisition Status

In order to analyze the pathogens identified as hazardous factors for prawns, a total of 958 samples were obtained, comprising 574 samples of seven species of crustaceans, 384 samples of eight species of mollusks, and 24 pooled samples of *Artemia franciscana* (as presented in Table 2).

### 3.2. Pathogen Detection

#### 3.2.1. Identification of Hazardous Factors

A hazard identification was conducted to evaluate diseases that have been associated with outbreaks in prawns. The diseases identified as potential hazards were subsequently analyzed to identify the specific pathogens involved and establish a disease surveillance program. In this study, pathogen screening was conducted for a total of twelve hazardous factors including CMNV, DIV1, EHP, IHHNV, IMNV, LSNV, NHP, TSV, AHPND, WSSV, WTD, and YHV1. The diseases that were subject to surveillance are detailed in Table S2.

#### 3.2.2. Prevalence of *Vp*_AHPND_ and WSSV

WSSV was detected in *Metapenaeus joyneri* and *Penaeus vannamei*, and the *Vp*_AHPND_ gene was also detected in some *P. vannamei* in which WSSV was detected. The prevalence of WSSV and *Vp*_AHPND_ is described in Table 3. Pathogens other than *Vp*_AHPND_ and WSSV were not detected.

#### 3.2.3. Viral Copies of WSSV

The viral genome copies of WSSV are distributed on average at 2.66 × 10^4^ copies/g (2.67 × 10 to 5.06 × 10^5^ copies/g) in the muscle and 5.84 × 10^5^ copies/g (3.77 × 10 to 1.04 × 10^7^ copies/g) in the midgut.

### 3.3. Infectiousness Assessment of Detected Pathogens

#### 3.3.1. Infectiousness Assessment of *Vp*_AHPND_

An attempt was made to isolate the pathogen from the tissue in which *Vp*_AHPND_ was detected, but it was presumed that the bacteria were not cultured and thus not a living pathogen, so an infectiousness assessment was not performed.

#### 3.3.2. Infectiousness Assessment of WSSV

There was no developed cell line in the case of the detected WSSV yet, so oral infection was attempted like in [19,20,21], but the infectiousness was not confirmed as in the [22] study results.

The experiment was conducted on seven segments, including a negative control for all different species in the six regions where each prawn was detected (to trace the origin of infectiousness). Except for one mortality in each of the 2nd and 5th segments on the 13th day of the experiment, no other mortalities occurred, and WSSV was not detected in any of the specimens after the experiment concluded. Twenty-five whiteleg shrimp (*P. vannamei*) were stocked per segment in a 300 L rectangular tank. The temperature was maintained at 24 ± 1 °C, dissolved oxygen at 8.0 ± 0.3 mg/L, salinity at 30 ± 0.4, and pH at 7.7 ± 0.1 throughout the experiment, which lasted for 14 days.

### 3.4. Histopathology

**Figure 1:**
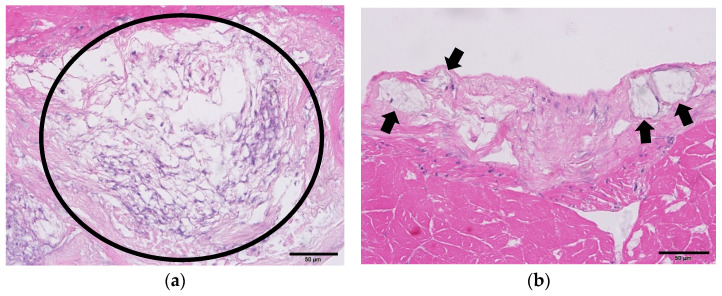
Histopathological results of the pathogen detected samples (**a**); necrotic lesions, presumed to be traces of inflammation (represented by a black circle), are observed in the *Vp*_AHPND_ infected sample (**b**); inclusion bodies are seen in presumed epithelial cells (indicated by black arrows) in the WSSV-infected sample. (H&E stain; scale bar = 50 μm).

**Figure 2:**
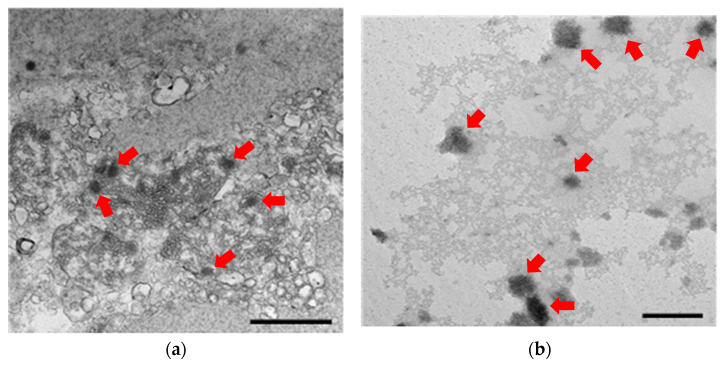
Ultramicroscopic results of the pathogen detected samples (**a**); WSSV-like particles are observed in the hamocyte (**left**; scale bar = 1 μm) (**b**); the sub-cuticular epithelial cell (**right**; scale bar = 200 nm) (indicated by red arrows).

Following histopathological analysis, evidence of necrosis indicative of inflammation was observed surrounding the sub-cuticular epithelial cells (Figure 1). Furthermore, transmission electron microscopy (TEM) analysis confirmed the presence of WSSV-like particles in the sub-cuticular epithelial cells (Figure 2). In contrast, no specific bacterial pathogen was observed in the *Vp*_AHPND_-infected sample.

## 4. Discussion

In this study, among 12 identified pathogens posing a threat to prawns, *Vp*_AHPND_ and WSSV were detected; however, the infectious nature of these pathogens was not confirmed. The failure to confirm infectivity is presumed to be due to the degradation of viral genes or envelope proteins caused by repeated freezing/thawing or prolonged storage under frozen conditions. Among the hazardous factors analyzed in this study, it has been reported that *Vp*_AHPND_ failed to cause infection after infected shrimp were frozen and re-infected [23]. In contrast, IHHNV maintained its infectivity even after repeated freezing/thawing cycles of infected tissues [24,25,26], and for WSSV, multiple studies have shown that it retains infectivity under freezing conditions ranging from −20 to −70 °C [27,28,29,30,31,32,33]. However, no infectivity was detected in the WSSV detected in this study. To demonstrate this, histopathological and ultramicroscopic analyses were performed, revealing severe tissue necrosis and the inability to determine the shape of *Vp*_AHPND_. Moreover, the particles suspected to be WSSV were not intact.

In this study, some cases of co-infection involving *Vp*_AHPND_ and WSSV were observed. Such co-infections are a common phenomenon in natural environments and can induce synergistic or antagonistic reactions within the host [34,35,36,37,38]. From the perspectives of quarantine and disease control, heightened management may be necessary in cases where interactions between endemic diseases prevalent domestically and those that could have entered from abroad are anticipated to be lethal to the hosts. In particular, co-infections involving pathogens known to inhibit host growth (such as IHHNV or EHP) and those capable of causing higher mortality rates (or prevalence) during juvenile stages (such as YHV1) can inflict significant damage on aquaculture facilities or natural environments [26,39,40].

Nevertheless, given the high proportion of detected cases and the timing of the sampling period coinciding with the global economic downturn caused by the COVID-19 pandemic, there is a risk of exposure to the domestic market while maintaining pathogenicity if the popularity of leisure activities such as fishing increases, leading to greater consumption and increased imports with a simplified distribution process. Therefore, despite the unconfirmed infectiousness, the risks associated with exposure to aquatic products (bait and feed) remain significant. Moreover, it is necessary to exercise greater caution with fishing bait or feed as they are directly exposed to the water, leading to the risk of disease transmission. In Australia, there have been cases of WSSV infection caused by imported shrimp intended for human consumption, underscoring the urgent need for measures to prevent misuse [7]. Furthermore, instances of disease transmission resulting from such changes in land use have been a consistent concern raised in recent years [3].

Particular attention is required for fishing (paste) bait, as it can directly transmit pathogens to susceptible hosts. Moreover, in South Korea, there are no separate processing standards or regulations for seafood products intended for bait or feed, which may lead to the possibility of infection through waste disposal. The importation of designated quarantine inspection materials is regulated by law, allowing imports only from places where there are frozen storage facilities for carcasses or waste (Article 35, Enforcement Regulation of the Aquatic Organism Disease Control Act). Therefore, it is necessary to establish policies that encourage the installation of such facilities not only in fishing gear stores but also in aquaculture facilities.

Repetitive freezing/thawing is an unavoidable process in the distribution of commercial seafood products and can also be considered as one of the methods for inactivating pathogens that can be presented as an imported hygiene condition. However, many regulatory/realistic issues need to be addressed to track or demonstrate freezing/thawing. Therefore, it may be possible to assume that there is no pathological activity for specific pathogens, by supporting the research results that state the loss of pathogenicity after a certain period of frozen storage, ranging from a few hours to several days, and proving that it has been stored frozen for a certain period. Such data will be important information for export/import inspections. Therefore, future studies will investigate the inactivation period of diseases identified as risk factors in this study under frozen conditions.

## 5. Conclusions

We detected *Vp*_AHPND_ and WSSV in fishing bait that was not subject to quarantine measures in South Korea. However, their infectivity was not confirmed, and this was presumed to be due to repeated freezing/thawing cycles or prolonged freezing/refrigeration storage. Histopathological and ultramicroscopic analysis revealed collapsed viral particles, which were suspected to be WSSV. Although infectivity was not confirmed in this study, the importation of aquatic products infected with pathogens without undergoing separate quarantine measures into South Korea is highly risky. Therefore, there is a need to perform quarantine measures on such non-quarantine-targeted aquatic products, and additional research is required to establish import hygiene conditions such as freezing and heating.

## Figures and Tables

**Table 1:** Sampling locations and origins of fishing bait analyzed in this study.

No.	Samples	Sampling Locations	Origins
1	*Metapenaeus joyneri*, *Portunus trituberculatus*, *Uroteuthis duvaucelii*	Jeonnam	China
2	*Eriocheir sinensis*	Jeonnam	China
3	*Perinereis aibuhitensis*, *Marphysa sanguinea*, *Metapenaeus joyneri*, *Euphausia superba*	Jeonnam	China, Antarctic Ocean
4	*Perinereis aibuhitensis*, *Loliolus beka*, *Todarodes pacificus*, *Amphioctopus fangsiao*, *Palaemon orientis*, *Euphausia superba*, *Metapenaeus joyneri*, *Penaeus vannamei*	Chungnam	Vietnam, Antarctic Ocean
5	*Loliolus beka*	Jeonnam	China
6	*Artemia franciscana*	Internet Market	China
7	*Perinereis aibuhitensis*, *Atrina pectinata*	Jeonbuk	China
8	*Artemia franciscana*	Chungnam	United States
9	*Todarodes pacificus*	Gyeongnam	China
10	*Penaeus vannamei*, *Euphausia superba*, *Perinereis aibuhitensis*	Jeju	China, Antarctic Ocean
11	*Marphysa sanguinea*, *Penaeus vannamei*, *Metapenaeus joyneri*	Gyeonggi	China
12	*Eriocheir sinensis*	Jeonbuk	China
13	*Perinereis aibuhitensis*, *Marphysa sanguinea*	Jeonnam	China
14	*Perinereis aibuhitensis*, *Marphysa sanguinea*, *Dosidicus gigas*, *Euphausia superba*	Kangwon	China, Argentina, Antarctic Ocean
15	*Metapenaeus joyneri*, *Perinereis aibuhitensis*	Gyeongbuk	China

**Table 2:** The species and quantity of fishing bait investigated for pathogens confirmed as hazardous factors in this study.

Phylum	Species and Quantity
Crustacean	11 of *Portunus trituberculatus*
	159 of *Metapenaeus joyneri*
	60 of *Eriocheir sinensis*
	210 of *Euphausia superba*
	30 of *Palaemon orientis*
	104 of *Penaeus vannamei*
	24 pooled samples * of *Artemia franciscana*
Semi-total	574 samples of 7 species + 24 pooled samples *
Mollusks	11 of *Uroteuthis duvaucelii*
	20 of *Loliolus beka*
	199 of *Perinereis aibuhitensis*
	57 of *Marphysa sanguinea*
	68 of *Todarodes pacificus*
	10 of *Amphioctopus fangsiao*
	1 of *Atrina pectinata*
	18 of *Dosidicus gigas*
Semi-total	384 animals of 8 species
Total	1556 samples of 37 species (+ 24 pooled samples *)

* In the case of *A. franciscana*, since the sample size was tiny, a pooled sample of 0.1 g per section was prepared.

**Table 3:** The detailed sampling regions, species, origins, and disease prevalence of samples with positive molecular biological test results for pathogens confirmed as hazardous factors in this study.

No.	Detailed Region	Species	Origin	Prevalence
1	Jeonnam Mokpo	*M. joyneri*	China	20% (6/30) of WSSV
2	Jeonnam Jindo	*P. vannamei*	Vietnam	100% (24/24) of WSSV
3	Jeju Hamduk	*P. vannamei*	Vietnam	100% (25/25) of WSSV and 28% (7/25) of *Vp*_AHPND_
4	Incheon Ganghwa	*M. joyneri*	Vietnam	26.7% (8/30) of WSSV
5	Incheon Ganghwa	*P. vannamei*	Vietnam	100% (25/25) of WSSV
6	Gyeongbuk Ulgin	*M. joyneri*	China	30% (9/30) of WSSV

## Data Availability

The datasets used and/or analyzed during the current study are available from the corresponding author on reasonable request.

## Supplementary Materials

The following supporting information can be downloaded at: https://www.mdpi.com/article/10.3390/pathogens12101228/s1, Table S1. Primers and PCR conditions used in this study; Table S2. Results of the hazard identification of fishing baits. References [41,42,43,44,45,46,47,48,49,50,51,52,53,54,55,56,57,58,59,60,61,62,63,64,65,66,67,68,69,70,71,72,73,74,75,76,77,78,79,80,81,82,83,84,85,86,87,88,89,90,91,92,93,94,95,96,97,98,99,100,101,102,103,104,105,106] are cited in the Supplementary Materials.
