# Supplementary material for: Identification of Potential Hazards Associated with South Korean Prawns and Monitoring Results Targeting Fishing Bait
Source: Pathogens. 2023 Oct 10;12(10):1228. doi: 10.3390/pathogens12101228 (PMC10610149; doi:10.3390/pathogens12101228)
Supplement: Supplementary file 1 [file pathogens-12-01228-s001.zip › pathogens-2617822-supplementary.pdf]

pathogens

MDPI

Table S1. Primers and PCR conditions used in this study.

Pathogen (or Disease)	Primers		Sequences (5′-3′)	Size (bp)	Conditions	Reference
Covert mortality nodavirus (CMNV)	Step 1	CMNV-7F1	AAA TAC GGC GAT GAC G	618	94 °C for 4 min, (94 °C for 30 s, 45 °C for 30 s, 72 °C for 40 s) × 35 cycles, 72 °C for 7 min	[14]
		CMNV-7R1	ACG AAG TGC CCA CAG AC			
	Step 2	CMNV-7F2	CAC AAC CGA GTC AAA CC	165	94 °C for 4 min, (94 °C for 20 s, 50 °C for 20 s, 72 °C for 20 s) × 30 cycles, 72 °C for 7 min	
		CMNV-7R2	GCG TAA ACA GCG AAG G			
Decapod iridescent virus 1 (DIV1)	Step 1	SHIV-F1	GGG CGG GAG ATG GTG TTA GAT	457	95 °C for 3 min, (95 °C for 30 s, 59 °C for 30 s, 72 °C for 30 s) × 35 cycles, 72 °C for 2 min	[15]
		SHIV-R1	TCG TTT CGG TAC GAA GAT GTA			
	Step 2	SHIV-F2	CGG GAA ACG ATT CGT ATT GGG	129	95 °C for 3 min, (95 °C for 30 s, 59 °C for 30 s, 72 °C for 20 s) × 35 cycles, 72 °C for 2 min	
		SHIV-R2	TTG CTT GAT CGG CAT CCT TGA			
Enterocytozoon hepatopenaei (EHP)	Step 1	SWP_1F	TTG CAG AGT GTT GTT AAG GGT TT	514	95 °C for 5 min, (95 °C for 30 s, 58 °C for 30 s, 68 °C for 45 s) × 30 cycles, 68 °C for 5 min	[41]
		SWP_1R	CAC GAT GTG TCT TTG CAA TTT TC			
	Step 2	SWP_2F	TTG GCG GCA CAA TTC TCA AAC A	148	95 °C for 5 min, (95 °C for 30 s, 64 °C for 30 s, 68 °C for 20 s) × 20 cycles, 68 °C for 5 min	
		SWP_2R	GCT GTT TGT CTC CAA CTG TAT TTG A			
		IHHNV389F	CGG AAC ACA ACC CGA CTT TA	389		[42]

Infectious hypodermal and haematopoietic necrosis virus (IHHNV)		IHHNV389R	GGC CAA GAC CAA AAT ACG AA		94 °C for 5 min, (94 °C for 30 s, 60 °C for 30 s, 72 °C for 30 s) × 35 cycles, 72 °C for 7 min	
Infectious myonecrosis virus (IMNV)	Step 1	4587F	CGA CGC TGC TAA CCA TAC AA	328	60 °C for 30 min, 95 °C for 2 min, (95 °C for 45 sec, 60 °C for 45 sec) × 39 cycles, 60 °C for 7 min	[43]
		4914R	ACT CGG CTG TTC GAT CAA GT			
	Step 2	4725NF	GGC ACA TGC TCA GAG ACA	139	95 °C for 2 min, (95 °C for 30 s, 65 °C for 30 s, 72 °C for 30 s) × 39 cycles, 72 °C for 2 min	
		4863NR	AGC GCT GAG TCC AGT CTT G			
Laem-Singh virus (LSNV)	Step 1	20AF	TTG CCT TCT CCC GAG TGG TC	200	94 °C for 5 min, (94 °C for 1 min, 60 °C for 1 min, 72 °C for 1 min) × 35 cycles, 72 °C for 10 min	[12]
		20AR	CCG GCT GAG GTA GCT GCT TG			
	Step 2	LSNVnF	GCG CAA GAG TTC TCA GGC TT	140	42 °C for 1 h, 94 °C for 5 min, (94 °C for 1 min, 60 °C for 1 min, 72 °C for 1 min) × 35 cycles, 72 °C for 10 min	[13]
		LSNVnR	ATC ACC GCA GGC TAA TAT AG			
Necrotising hepatopancreatitis (NHP)		NHPF2	CGT TGG AGG TTC GTC CTT CAG T	379	95 °C for 5 min, (95 °C for 30 s, 60 °C for 30 s, 72 °C for 30 s) × 35 cycles, 60 °C for 1 min, 72 for 2 min	[44]
		NHPR2	GCC ATG AGG ACC TGA CAT CAT C			
Taura syndrome virus (TSV)		9992F	AAG TAG ACA GCC GCG CTT	231	60 °C for 30 min, 94 °C for 2 min,	[30]
		9195R	TCA ATG AGA GCT TGG TCC			

					(94 °C for 45 s,<br>60 °C for 45 s)<br>× 40 cycles,<br>60 °C for 7 min	
Vibrio parahaemolyticus containing Pir toxins (*Vp*AHPND)	Step 1	AP4-F1	ATG AGT AAC AAT ATA AAA CAT GAA AC	1269	94°C for 2 min,<br>(94°C for 30 s,<br>55°C for 30 s,<br>72°C for 90 s)<br>x 30 cycles,<br>72°C for 2 min	[45]
		AP4-R1	ACG ATT TCG ACG TTC CCC AA			
	Step 2	AP4-F2	TTG AGA ATA CGG GAC GTG GG	230	94 °C for 2 min,<br>(94 °C for 20 s,<br>55 °C for 20 s,<br>72 °C for 20 s)<br>× 25 cycles	
		AP4-R2	GTT AGT CAT GTG AGC ACC TTC			
White spot syndrome virus (WSSV)	Step 1	146F1	ACT ACT AAC TTC AGC CTA TCT AG	1447	94 °C for 4 min,<br>55 °C for 1 min,<br>72 °C for 2 min,<br>(94 °C for 1 min,<br>55 °C for 1 min,<br>72 °C for 2 min)<br>× 39 cycles,<br>72 °C for 5 min	[46]
		146R1	TAA TGC GGG TGT AAT GTT CTT ACG A			
	Step 2	146F2	GTA ACT GCC CCT TCC ATC TCC A	941		
		146R2	TAC GGC AGC TGC TGC ACC TTG T			
White tail disease (WTD)	Step 1	MrNV-F	GAT ACA GAT CCA CTA GAT GAC C	681	52 °C for 30 min,<br>95 °C for 2 min,<br>(94 °C for 40 s,<br>55 °C for 40 s,<br>68 °C for 1 min)<br>× 30 cycles	[47]
		MrNV-R	GAC GAT AGC TCT GAT AAT CC			
	Step 2	XSV-F	GGA GAA CCA TGA GAT CAC G	500		
		XSV-R	CTG CTC ATT ACT GTT CGG AGT C			
Yellow head virus genotype 1 (YHV1)	Step 1	YC-F1ab	ATC GTC GTC AGY TAY CGY AAY ACY GC	358	50 °C for 30 min,<br>94 °C for 2min,<br>95 °C for 15 min,<br>(94 °C for 45 s,<br>60 °C for 45 s,<br>68 °C for 45 s)<br>× 35 cycles,<br>68 °C for 7 min	[48]
		YC-R1ab	TCT KCR YGT GTG AAC ACY TTC TTR GC			
	Step 2	YC-F2ab	CGC TTY CAR TGT ATC TGY ATG CAC CA	146	95 °C for 15 min,<br>(94 °C for 45 s,<br>60 °C for 45 s,	
		YC-R2ab	RTC DGT GTA CAT RTT DGA GAG TTT RTT			

		72 ℃ for 45 s)<br>× 35 cycles,<br>72 ℃ for 7 min

Table S2. Results of the hazard identification of fishing baits.

Pathogen (Disease)	Susceptible species	Reportable or listed disease (Yes/No)		Geographic distribution		Sub-types	Zoonotic disease (Yes/No)	Entry may cause an outbreak and increase domestic damage (Yes/No)	Establishment and implementation of a monitoring plan at the national level (Yes/No)	Considered a hazard in this study? (Yes/No: Reasons)	References
		**WOAH**	**South Korea**	**Others**	**South Korea**						
Vibrio parahaemilyticus strains containing Pir toxins *Vp*AHPND (acute hepatopancreatic necrosis disease, AHPND)	*Penaeus chinensis* *Penaeus japonicus* *Penaeus monodon* *Penaeus semisulcatus* *Penaeus vannamei* (various penaeid species)	Yes	Yes	Bangladesh China Costa Rica Egypt Malaysia Mexico Myanmar Peru Philippines Taiwan Thailand Vietnam	Yes	-	No	Yes	Yes	Yes: AHPND is listed by the WOAH and is associated with significant losses in prawn farming environment and is widespread in countries likely to export large quantities of prawns to Korea. AHPND is also included on the *List of diseases in Korea*, and the *List of diseases in the Asia-Pacific*.	[49–56]

	AHPND means infection with strains of *Vibrio parahaemolyticus* (*Vp*AHPND) that contain a ~70 kbp plasmid with genes that encode homologues of the *Photorhabdus* insect-related (Pir) toxins, PirA and PirB. Although there are reports of the isolation of other *Vibrio* sp. from clinical cases of AHPND, only *Vp*AHPND has been demonstrated to cause AHPND. This pathogenic agent complies with the criteria described in the *WOAH Aquatic Animal Health*

									Code Article 2.1.2. Hazard Identification and will be retained for risk assessment.	
Candidatus Hepatobacter penaei (Necrotising hepatopancreatitis, NHP)	*Penaeus aztecus* *Penaeus duorarum* *Penaeus marginatus* *Penaeus merguiensis* *Penaeus setiferus* *Penaeus stylirostris* *Penaeus vannamei* (various penaeid species) *Penaeus monodon* (experimental infection only) *Homarus americanus* (PCR result only)	Yes	Yes	Americas Malaysia Thailand USA Vietnam	No	No	No	Yes	Yes	Yes: NHP is WOAH listed and associated with significant losses in prawn farming environment and is widespread throughout the world. NHP is included on the *List of diseases in Korea* and the *List of diseases in the Asia-Pacific*. This pathogenic agent complies with the criteria described in the *WOAH Aquatic Animal Health Code Article 2.1.2. Hazard Identification*	[57]

										and will be retained for risk assessment.	
Enterocytozoon hepatopenaei (EHP)	*Penaeus japonicus* *Penaeus monodon* *Penaeus stylirostris* *Penaeus vannamei* (various penaeid species)	No	Yes	Asia Venezuela	Yes	No	No	Yes		Yes: EHP is associated with significant disease in Asia and is included on the *List of diseases in Korea* and *List of diseases in the Asia-Pacific*. This pathogenic agent complies with the criteria described in the *WOAH Aquatic Animal Health Code Article 2.1.2. Hazard Identification* and will be retained for risk assessment.	[58–65]
Covert mortality nodavirus (CMNV)	*Macrobrachium rosenbergii* *Penaeus chinensis*	No	No	China Ecuador Thailand Vietnam	No	No	No	Uncertain	No	Yes: CMNV has caused serious losses in China and cumulative mortalities of	[14,66–68]

	Penaeus japonicus *Penaeus monodon* *Penaeus vannamei…* (various penaeid and caridean species) *Mugilogobius abei* *Paralichthys olivaceus* (finfish)									up to 80-90% of *Penaeus vannamei* in culture. CMNV is not included on the *List of diseases in Korea*. CMNV is included in the *List of Diseases in the Asia-Pacific*. This pathogenic agent complies with the criteria described in the *WOAH Aquatic Animal Health Code 2.1.2. Hazard Identification* and will be retained for risk assessment.	
Decapod iridescent virus 1 (DIV1) or *Cherax quadricarinatus* iridovirus (CQIV) or Shrimp	*Cherax quadricarinatus* *Macrobrachium nipponense* *Macrobrachium rosenbergii*	Yes	Yes	China Indian Ocean Taiwan	No	No	No	Yes	Yes	Yes: the National Fishery Products Quality Management Service (NFQS)	[15,69–76]

hemocyte iridescent virus (SHIV)	*Penaeus chinensis* *Penaeus japonicus* *Penaeus monodon* *Penaeus vannamei* *Procambarus clarkii…* (various penaeid and caridean species) *Cladocera* sp. Polychaetes (aquatic animals) *Exopalaemon carinicauda* *Pachygrapsus crassipes* *Eriocheir sinensis* (experimental infection only)		notes that although there is limited information regarding DIV1, it is considered a serious emerging disease in aquaculture in China and appears to be spreading throughout the surroundings of farming areas in China - large volumes of imported prawns are sourced from areas that may be affected by DIV1. Recent reports indicate it may be present in Thailand. Complete genome sequencing has

	revealed that CQIV and SHIV are different strains or genotypes of the same virus. The genome of SHIV was shown to be 99% identical to the genome of CQIV. Recently, SHIV and CQIV were formally classified by the International Committee on Taxonomy of Viruses (ICTV) under the name Decapod iridescent virus 1 (DIV1) in the family Iridoviridae (ICTV, 2018). DIV1 is included in *the List of diseases in the Asia-Pacific*, and listed as a

										disease notifiable to the WOAH and in the *List of diseases in Korea*. This pathogenic agent complies with the criteria described in the *WOAH Aquatic Animal Health Code Article 2.1.2. Hazard Identification* and will be retained for risk assessment.	
Infectious hypodermal and haematopoietic necrosis virus (IHHNV)	*Macrobrachium rosenbergii Penaeus californiensis Penaeus monodon Penaeus setiferus Penaeus stylirostris Penaeus vannamei…* (various penaeid and	Yes	Yes	Africa Asia America Pacific islands	Yes	Multiple strains	No	Yes	Yes	Yes: is present in Korea. IHHNV is listed by WOAH, is listed on *List of diseases in Korea*.	[77–80]

	caridean species)									
Infectious myonecrosis virus (IMNV)	*Penaeus esculentus* *Penaeus merguiensis* *Penaeus monodon* *Penaeus vannamei*… (various penaeid species) *Artemia franciscana* *Penaeus stylirostris* *Penaeus subtilis* (experimental infection only)	Yes	Yes	Brazil Burma China India Indian Ocean Indonesia Malaysia Myanmar	Yes	No	No	Yes	Yes	Yes: IMNV is WOAH listed and has been responsible for considerable losses in the Brazilian prawn farming industry and is present in Asia. IMNV is included on the *List of diseases in Korea* and the *List of diseases in the Asia-Pacific*. This pathogenic agent complies with the criteria described in the *WOAH Aquatic Animal Health Code Article 2.1.2. Hazard Identification* and will be retained for risk assessment.	[81–83]

Laem-Singh virus (LSNV) (Wenzhou shrimp virus genotpye 9, WZSV9) (Monodon slow growth syndrome, MSGS)	*Penaeus dobsoni Penaeus merguiensis Penaeus monodon Penaeus vannamei…* (various penaeid species)	No	No	China India Indonesia Malaysia Philippines Sri Lanka Thailand Vietnam	No	No	No	Yes	No	Yes: It has recently been determind that LSNV and WZSV9 are different isolates of the same virus species. Although there is limited information regarding LSNV and its role in MSGS, large volume of imported prawns are sourced from countries that may be affected by MSGS. LSNV will be considered in context with MSGS. This pathogenic agent complies with the criteria described in the *WOAH Aquatic Animal Health Code Article*	[13,84–88]

										2.1.2. Hazard Identification and will be retained for risk assessment.	
Taura syndrome virus (TSV)	*Penaeus aztecus* *Penaeus ensis* *Penaeus indicus* *Penaeus monodon* *Penaeus setiferus* *Penaeus stylirostris* *Penaeus vannamei…* (various penaeid and caridean species) *Penaeus merguiensis* *Macrobrachium rosenbergii* (experimental infection only)	Yes	Yes	Americas (including Hawaii) China East Africa Indonesia Malaysia Middle East Myanmar Taiwan Thailand Vietnam	Yes	Yes	No	Yes	Yes	Yes: TSV is WOAH Listed and is associated with significant losses in prawn farming environment and is widespread throughout the world. TSV is included on the *List of diseases in Korea*, and the *List of diseases in the Asia-Pacific*. This pathogenic agent complies with the criteria described in the *WOAH Aquatic Animal Health Code Article 2.1.2. Hazard Identification*	[26,89–90]

									and will be retained for risk assessment.	
White spot syndrome virus (WSSV)	All decapod Crustaceans from marine, brackish or freshwater sources challenged with infection with WSSV are susceptible	Yes	Yes	Americas Asia East Africa Middle East	Yes	No	No	Yes	Yes	Yes: WSSV is WOAH listed and associated with significant losses in prawn farming environment and is widespread throughout the world. WSSV is included on the *List of diseases in Korea*, and the *List of diseases in the Asia-Pacific*. Korea is managing an outbreak of WSSV. This pathogenic agent complies with the criteria described in the *WOAH Aquatic Animal Health Code Article 2.1.2. Hazard*	[91–96]

										Identification and will be retained for risk assessment.	
Macrobrachium rosenbergii nodavirus (MrNV) and extra small virus (XSV) or *Macrobrachium* muscle virus (MMV) (White tail disease, WTD)	*Macrobrachium rosenbergii* *Penaeus indicus* *Penaeus monodon* *Penaeus japonicus* *Penaeus vannamei*… (various penaeid and caridean species)	Yes	Yes	China Dominican Republic French West Indies India Indonesia Malaysia Myanmar Taiwan Thailand Vietnam	Yes	No	No	Yes	Yes	Yes: present in Korea but subject to control or eradication. WTD is included on the *List of diseases in Korea, WOAH,* and *the List of diseases in the Asia-Pacific*.	[97–103]
Yellow head virus genotype 1 (YHV1)	*Penaeus monodon* *Penaeus stylirostris* *Penaeus vannamei* (various penaeid and caridean species) *Palaemonetes pugio*	Yes	Yes	Egypt Indonesia Malaysia Mexico Myanmar Philippines Sri Lanka Taiwan Thailand	Yes	Genotype 1-10	No	Yes	Yes	Yes: YHV1 is WOAH listed and has been found in many commercially important wild and cultured species throughout the world at relatively high prevalence and is increasingly	[104–106]

Metapenaeus affinis (experimental infection only)	being associated with co-infections and stunted growth. Infection with YHV1 is included on the *List of diseases in Korea*, and the *List of diseases in the Asia-Pacific*. This pathogenic agent complies with the criteria described in the *WOAH Aquatic Animal Health Code Article 2.1.2. Hazard Identification* and will be retained for risk assessment.

4
